# Effect of Polyethylene Glycol Methyl Ether Methacrylate on the Biodegradability of Polyvinyl Alcohol/Starch Blend Films

**DOI:** 10.3390/polym15153165

**Published:** 2023-07-26

**Authors:** Asylzat Iskalieva, Mateyev Yesmurat, Khaldun M. Al Azzam, Dana Ainakulova, Yerzhanov Yerbolat, El-Sayed Negim, Mohamad Nasir Mohamad Ibrahim, Yeligbayeva Gulzhakhan

**Affiliations:** 1 School of Chemical Engineering, Kazakh-British Technical University, Str. Tole bi, 59, Almaty 050000, Kazakhstan; 2 «LF COMPANY» LLP, Zhambyl Region, Village Named after B. Momyshuly, Zhibek Zholy Str., 3b, Almaty 080300, Kazakhstan; mateew@mail.ru; 3 Pharmacological and Diagnostic Research Center (PDRC), Department of Pharmaceutical Sciences, Faculty of Pharmacy, Al-Ahliyya Amman University, Amman 19328, Jordan; azzamkha@yahoo.com; 4 School of Materials Science and Green Technologies, Kazakh-British Technical University, St. Tole bi, 59, Almaty 050000, Kazakhstan; dana.ainakulova@gmail.com (D.A.); elashmawi5@yahoo.com (E.-S.N.); 5 School of Petroleum Engineering, Satbayev University, 22 Satpayev Street, Almaty 050013, Kazakhstan; gulzhakh@yandex.ru; 6 School of Chemical Sciences, Universiti Sains Malaysia, Pulau Pinang 11800, Malaysia; mnm@usm.my

**Keywords:** grafting, starch, polyvinyl alcohol, blend films, solubility, transparency, biodegradability

## Abstract

Blend copolymers (PVA/S) were grafted with polyethylene glycol methyl methacrylate (PEGMA) with different ratios. Potassium persulfate was used as an initiator. The blend copolymer (PVA/S) was created by combining poly(vinyl alcohol) (PVA) with starch (S) in various ratios. The main idea was to study the effect of different ratios of the used raw materials on the biodegradability of plastic films. The resulting polymers (PVA/S/PEGMA) were analyzed using FTIR spectroscopy to investigate the hydrogen bond interaction between PVA, S, and PEGMA in the mixtures. TGA and SEM analyses were used to characterize the polymers (PVA/S/AA). The biodegradability and mechanical properties of the PVA/S/PEGMA blend films were evaluated. The findings revealed that the mechanical properties of the blend films are highly influenced by PEGMA. The time of degradation of the films immersed in soil and Coca-Cola increases as the contents of PVA and S and the molecular weight (MW) of PEGMA increase in the terpolymer. The M8 sample (PVA/S/PEGMA in the ratio of 3:1:2, respectively) with a MW of 950 g/mol produced the lowest elongation at break (67.5%), whereas M1 (PVA/S/PEGMA in the ratio of 1:1:1, respectively) with a MW of 300 g/mol produced the most (150%). The film’s tensile strength and elongation at break were improved by grafting PEGMA onto the blending polymer (PAV-b-S). *T*_g_ and *T*_m_ increased when the PEGMA MW increased from 300 to 950. *T*_g_ (48.4 °C) and *T*_m_ (190.9 °C) were the lowest in M1 (300), while *T*_g_ (84.8 °C) and *T*_m_ (190.9 °C) were greatest in M1 (950) at 209.3 °C. The increased chain and molecular weight of PEGMA account for the increase in *T*_g_ and *T*_m_ of the copolymers.

## 1. Introduction

The physico-mechanical characteristics and economic viability of synthetic polymers manufactured from polyethylene, polypropylene, and polystyrene have led to their widespread use as packing materials. However, the non-biodegradable, petroleum-based polymers create a problem of plastic trash utilization, and their amount increases every year [1,2,3]. It has been shown that the usage of biodegradable polymers, which readily break down into carbon dioxide, water, methane, inorganic compounds, and biomass when subjected to environmental physical variables and microbial activity, is the ideal method for resolving these issues [4,5].

Natural polymers, such as starch, cellulose, protein, and chitin, are a more attractive option due to their biodegradability and renewable qualities. They have been thought of as replacements for petroleum-based and non-biodegradable polymers [3,4,5,6].

There are numerous projects devoted to developing biodegradable polymeric materials that can be used as packaging materials. The majority agree that starch is the best biomaterial because of its availability, affordability, and superior film-forming properties [7].

The hydrophilic characteristics and poor mechanical properties of starch-based films limit their usage [3,8]. By adding various plasticizers, such as glycerol, urea, sorbitol, formamide, etc., numerous attempts have been made to improve the mechanical and tensile properties of the films [1,2,3,4,5,6,7,8,9]. The tensile strength and elastic modulus of the modified starch-based films have been enhanced by the use of nano-size perspective fillers. On this route, naturally occurring polymer nanocrystals [10,11], micro-fibrillated cellulose [12], clays [13], carbon nanotubes [14], graphene oxide [15], etc. are used to enhance starch-based thermoplastic composites.

One more method has been assigned to reduce the drawbacks of starch-based materials. Starch and biodegradable polymers together greatly enhance the material’s physical characteristics [3]. For instance, adding poly (lactic acid), poly (butylene succinate), and polycaprolactone to starch-based composites has shown an improvement in their strength and water resistance [3].

Due to its exceptional qualities—including a high oxygen barrier, high strength, superior chemical resistance, excellent film-forming efficiency, significant water solubility, and other attributes—polyvinyl alcohol (PVA) has been widely used as a material for packaging [16,17,18,19]. PVA crystallites serve as crosslinking centers in the polymer matrix [20].

The utilization of PVA’s hydroxyl groups frequently involves the crosslinking process. To produce the reaction and form hydrogen bonds, a crosslinking agent is employed [21]. For the creation of PVA/starch (S) blend films, Tian et al. [3] used formaldehyde, glutaraldehyde, acetaldehyde, tetra ethyl ortho silicate, and other mono aldehydes as crosslinking agents.

Tian et al. [3] created films from a PVA/S combination of varied compositions. Melt processing was used in the procedure. The effect of the composition and relative humidity (RH) on the structure and properties of the resultant mixes was investigated and analyzed. Hydrogen bond interactions are formed by the OH hydroxyl groups in S and PVA. These links improve the two components’ compatibility. The crystallinity of the resulting component and the water uptake at equilibrium decreased as the starch content increased. The mechanical properties also decreased with an increase in the amount of starch. With a starch content of 50% in the PVA component, the flexibility of the blended films was high, the elongation at break was 1000%, and the tensile strength was 9 MPa. These data indicate that the resulting films have better properties than conventional LDPE packaging films. Thus, it was concluded that mixed PVA/S films could be an alternative material as packaging materials.

Fahmy et al. studied the synthesis of PVA and dimethoxydimethylsilane (DMDMS), which resulted in forming an approximately 17–30% crosslinked reinforced SiOC linkage within the network of composite membranes [22,23].

Negim et al. [24] used glacial acetic acid as a crosslinking agent, which improved the biodegradability of PVA/starch blend films. The physical, thermal, and mechanical properties of PVA/S mixtures were analyzed with a change in the ratio of the mixtures and the molecular weight of the PVA. In addition, the biodegradability of blended films was studied. The findings demonstrated that the PVA content and molecular weight in the PVA/S mixes had a significant impact on the physical and mechanical properties. Also, it was discovered that blended PVA/S films had noticeably increased biodegradability, particularly in moist soil. Later, Negim et al. [25] modified the PVA/S blend films using grafting polymerization of acrylic acid (AA) on the blend films at different concentrations (0.125, 0.25, and 0.50% by mass of PVA/S). FTIR, TGA, DTG, SEM, and mechanical tests were used to characterize the produced copolymers (PVA/S/AA). The results demonstrated that the grafting process improved the mechanical qualities of the blend films. The thermal stability, tensile strength, and elongation at break all increased with an increase in the AA ratio in the grafted copolymers.

Although there are a large number of works devoted to the creation of biodegradable polymeric materials, the problem of creating biodegradable systems to reduce the time of biodegradability has not yet been completely solved. This study aims to evaluate the effect of PEGMA on the biodegradability of PVA/S/PEGMA films, which have the potential to replace conventional packaging, and these films are suggested to be used for goods packaging.

## 2. Experimental

### 2.1. Materials

The corn starch (S) was obtained from “Everest” Ltd. (Moscow, Russia). Polyvinyl alcohol (PVA) with a molecular weight of 70,000–100,000 g/mol was supplied by Sigma-Aldrich Company (St. Louis, MO, USA). Polyethylene glycol methyl methacrylate (PEGMA) with varied molecular weights (300, 500, and 950 g/mol) was purchased from Sigma-Aldrich Company (USA). Potassium persulfate (PPS) was purchased from Merck (Darmstadt, Germany). All these chemicals were used without purification.

### 2.2. Methods

The research was conducted where polyvinyl alcohol (PVA) was mixed with starch (S) and Poly(ethylene glycol) methyl ether methacrylate (PEGMA).

#### 2.2.1. Synthesis of the Blend Copolymer

Different methods for the synthesis of biodegradable polymer composites were found in the literature [3,4,5]. In our study, the solutions of the PVA/S blend in various ratios were prepared using a procedure similar to that recently reported [24]. Briefly, 5 g PVA was dissolved in 100 mL distilled water using 500 mL three-necked flasks. After that, it was stirred under mechanical stirring and heated in a water bath at 50 °C for 30 min until the PVA was completely dissolved. Then, 5 g starch (S) was dissolved in 50 mL distilled water and then added to the three-necked flasks with the obtained PVA solution. After that, the mixture was heated at 70 °C for another 4 h while stirring at 800 rpm until the PVA completely dissolved. The details of the blend copolymer’s PVA and S mix ratios are summarized in Table 1.

#### 2.2.2. Synthesis of the Grafting Polymer

Potassium persulfate was prepared by dissolving 0.1 g in 10 mL distilled water while stirring for 10 min at 70 °C. Then, it was added to the PVA/S solutions. The PEGMA monomer with varied molecular weights (300, 500, and 950 g/mol) and proportions (5, 7, 10, and 15 g) was added to the previous solutions (M1- 1:1, M2- 1.5:1, M3- 2:1, M4- 3:1, M5- 3:1.5, M6- 3:2, M7- 3:1, and M8- 3:1) drop-by-drop for 1 h during the stirring process at 70 °C using an automatically controlled water bath under a nitrogen atmosphere (Table 2). After that, the reaction was allowed to proceed for another 3 h at 70 °C.

#### 2.2.3. Preparation of the PVA/S/PEGMA Films

The casting solution technique was used to obtain the PVA/S/PEGMA films in a highly cleaned glass dish. The process for the preparation of the PVA/S/PEGMA films involved pouring their aqueous solutions onto flat glass surfaces and allowing them to dry at room temperature (25 °C) with a relative humidity of 59% for 7 days followed by an aerated oven at 60 °C for 12 h to entirely remove the water [24,25]. To remove any remaining PEGMA, the films were extensively washed with distilled water and then acetone. The films were then dried and kept at room temperature in a desiccator for additional characterization and measurements.

#### 2.2.4. Tests

The synthesized polymers were subjected to FTIR studies using an infrared spectrophotometer made by Bruker, model Alpha. The spectra were recorded from 4000 to 400 cm^−1^, where the resolution was 4 cm^−1^ with an average of 16 scans. Thermogravimetric analysis (TGA) measurements were performed using a TGA/SDTA851e from METTLER TOLEDO. The experiments were conducted at a heating rate of 10 °C min^−1^ between 30 and 900 °C. TGA shows the thermostability and temperature of degradation.

According to the ASTM D 882-91 [26], the tensile strength and elongation at break of the films were measured using the MTS 10/M tensile testing machine with a crosshead speed of 50 mm/min. The tensile characteristics of the mixed films were evaluated. A minimum of four readings were averaged, and the 1-kN load cell was employed. Scanning electron microscopy (SEM) images on a Carl Zeiss SMT, Oberkochen were used to examine the microstructure of the hydrophilic polymers. SEM was in operation at an acceleration voltage of 15 kV. Magnification power varied from 500 to 2000.

The sample means and standard deviation (SD) were calculated to examine the results using two common descriptive techniques. The mean was calculated by summing up all the data points and dividing them by the number of tests that were run in triplicate. The standard deviation, which reflects the positive square root of the variance, is a fundamental statistical analytical tool. It quantifies how varied or dispersed the dataset is. If the standard deviation is low, the data are likely to be closely grouped around the mean, whereas if it is large, the data are more likely to be spread out from the mean.

## 3. Results and Discussion

**Figure 1:**
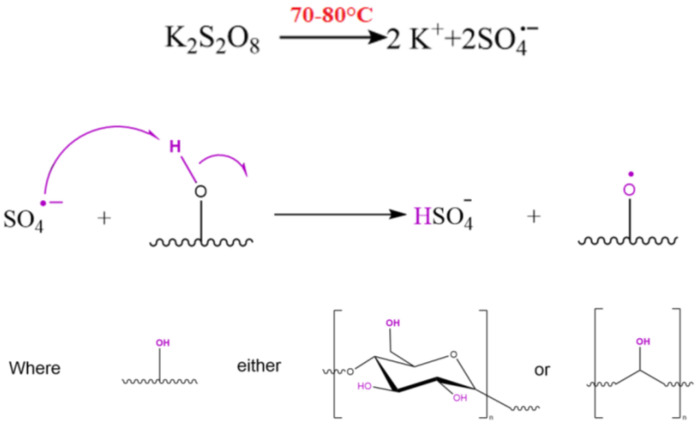
The mechanism of initiation of potassium persulfate.

Potassium persulfate is decomposed at 70 °C, forming an anion radical. Then, hydrogen transfers from the hydroxyl group of the polymers (either PVA or S), activating oxygen [27]. The mechanism of initiation of potassium persulfate is shown in Figure 1.

**Figure 2:**
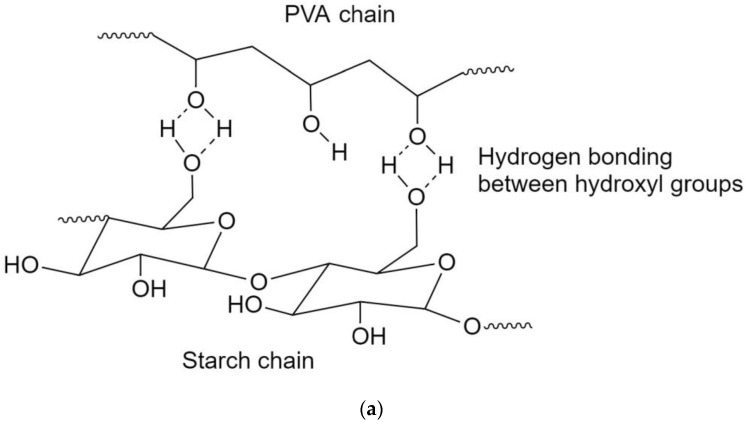

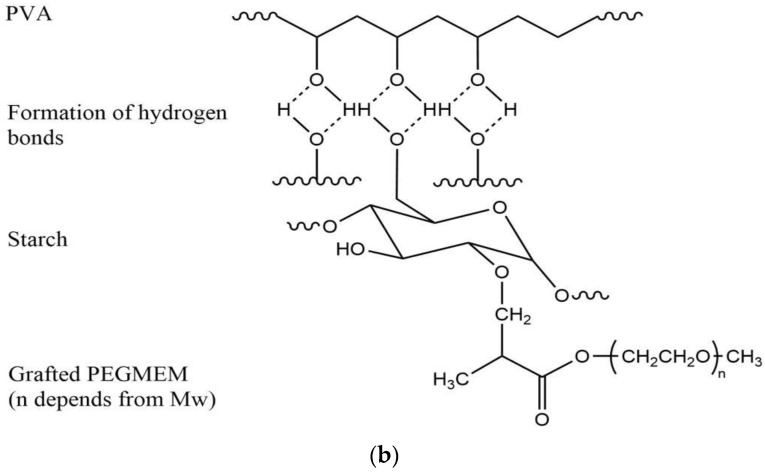
(**a**) Hydrogen bonding between PVA and starch chains. (**b**) The chemical structure of the (PVA/S)-g-PEGMA formed.

Both PVA and starch have an excess of hydroxyl groups; they interact favorably and create both intra- and inter-molecular hydrogen bonds (Figure 2a). The chemical structure of the (PVA/S)-g-PEGMA formed is shown in Figure 2b.

### 3.1. FTIR Spectra

**Figure 3:**
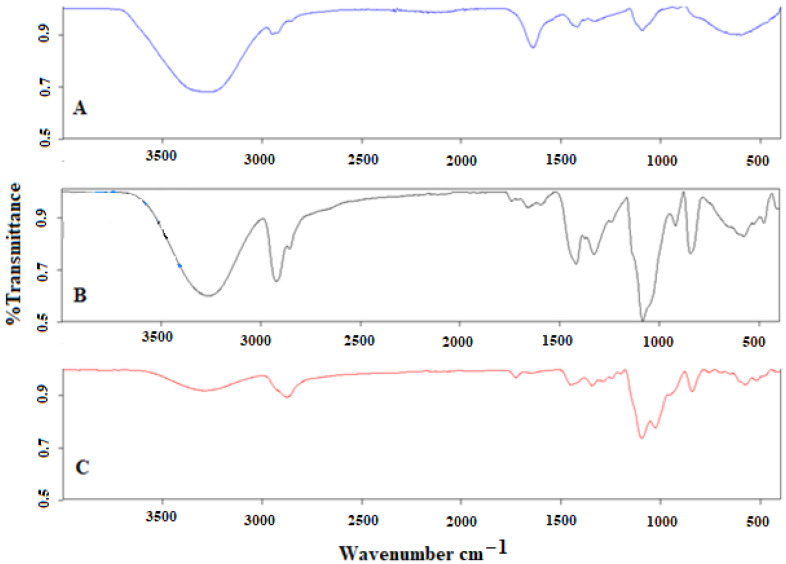
The FTIR spectra of (**A**) PVA, (**B**) PVA/S, and (**C**) (PVA/S)-g-PEGMA.

Figure 3 shows the FTIR spectra of PVA, the PVA/S blend, and (PVA/S)-g-PEGMA.

The intensity of the bands at 3382, 3234, and 3247 cm^−1^ was reduced in (PVA/S)-g-PEGMA. This reduction can be noted as the intramolecular hydrogen bonding formed from a novel interaction between the OH groups in starch and PVA [28].

In Figure 3A, peaks at 2941, 1239, 919, 1097, and 3382 cm^−1^ can be observed, which are attributed to the C–H stretching, C–H bending, C–O stretching, and O–H stretching bands of PVA and starch, respectively, while the peak at 1708 cm^−1^ is due to the carbonyl group for the residual acetic groups during the manufacturing of PVA from polyvinyl acetate using the hydrolysis process [24,29,30,31,32]. Figure 3B demonstrates new bands at 1240, 1082, and 1020 cm^−1^ that are related to the stretching of C–O in the C–O–H groups and the C–O stretching of the vibration of the C–O–C bonds, which confirms the crosslinking reactions [28,29,30,31,32,33]. The intensity of the band at 1636 cm^−1^ can be signified as the carbonyl group [31].

Figure 3C displays new bands at 2942, 1242, 1080, 928, and 3247 cm^−1^ that are related to the C–H, C–H, C–O stretching and O–H stretching bands [19,24,29]. The band at 1712 cm^−1^ belongs to the C=O carbonyl group, and the intensity of this band decreases with the functionalization process of PVA/S/PEGMA [31].

### 3.2. Thermogravimetric Analysis (TGA)

Thermogravimetric analysis (TGA) of the polymers is conducted to measure weight changes as a function of temperature and time. The weight changes in polymeric materials can be caused by decomposition and oxidation reactions as well as physical processes, such as sublimation, vaporization, and desorption.

The thermal stability of the copolymer films of (PVA-b-S)-g-PEGMA with composition ratio 1:1:1 (M1) and different MWs of PEGMA was evaluated using TGA at temperature ranges from 29 to 900 °C at 25 °C/min in air. Table 3 presents the stage steps, temperature range, residue (%), weight loss (%), initial degradation temperature (IDT), and the maximum polymer degradation temperature (PDT_max_). Thermal degradation for M1 (300) proceeds in three steps, but for M1 (500) and M1 (950), thermal degradation proceeds in five steps. The first degradation step for M1 (300) occurs at a temperature range from 29.7 to 386.5 °C, while for M1 (500), it occurs at a range from 30.7 to 121.8 °C, and for M1 (950), it occurs at a range from 29.8 to 219.1 °C. The weight loss percentage for the first degradation step was 87.4 for M1 (300), 8.4 for M1 (500), and 14.7 for M1 (950) due to the loss of moisture, water, and solvent. The weight loss percentage for the second degradation stage for M1 (300) begins at 386.5 °C with 4.4% lost, for M1 (500) occurs at 121.8 °C with 10.6% lost, and for M1 (950) occurs at 219.1 °C with 4.5% lost. This is attributed to the decomposition of starch and loss of CO_2_. In the third stage, in the temperature range 531.6–896.9 °C, there was a weight loss of 14.6% for M1 (300); in the temperature range 182.8–261.1 °C, there was a weight loss of 25.8% for M1 (500); and in the temperature range 275.7–333.3 °C, there was a weight loss of 5.6% for M1 (950), owing to the decomposition of PVA. The fourth and fifth degradation stages occur between 261.1 and 524.3 °C with a weight loss range from 25.3 to 4.8% for M1 (500) and occur between 333.3 and 899.2 °C with a weight loss range from 14.2 to 20.3% for M1 (950) due to the decomposing of active groups, such as carbonyl, ether, and hydrogen bonds. It can be observed that IDT and PDT_max_ increase with an increase in the molecular weight of PEGMA. The increase in IDT and PDT_max_ is due to the increasing molecular weight of PEGMA from 500 to 950 [34,35,36,37].

Table 3 shows the *T*_g_ and *T*_m_ of the copolymers with different molecular weights. It was observed that the *T*_g_ and *T*_m_ increased with an increase in the molecular weight of PEGMA from 300 to 950. M1 (300) gave the lowest *T_g_* (48.4 °C) and *T_m_* (190.9 °C), while M1 (950) gave the highest *T_g_* (84.8 °C) and *T_m_* (209.3 °C). The increase in the *T_g_* and *T_m_* of the copolymers is attributed to the increasing chain and molecular weight of PEGMA.

Surprisingly, TGA displays weight changes as a function of temperature or time. This usually refers to how much weight was lost while heating (in mg or weight %). However, mass gain during TGA tests can happen under specific circumstances, such as when an oxidizing agent, water molecules, or impurities are present in the sample, and upon heating to 900 °C, the complete degradation will not be 100%. This is what had been observed for the samples being analyzed [38].

Two important characteristics that are utilized to comprehend how polymers behave are the *T*_g_ and *T*_m_. Glass transition temperature is abbreviated as *T*_g_, whereas melting temperature is abbreviated as *T*_m_. The polymer’s characteristics dictate how strong and elastic it is. The molecular weight, flexibility, and crystallinity of the polymer are only a few of the many factors that affect the polymer’s properties. We can better understand how various polymers react in various circumstances by being aware of their features. Because a polymer might have different melting points because it contains several components with various molecular weights or structures, the *T*_g_ is often lower than the *T*_m_, and the polymer is rigid at ambient temperature. It does not have a distinct melting point if the *T*_g_ is higher than the *T*_m_, and heating will reveal this.

Additionally, mechanical degradation reduces the average molecular weight of the polymer. Although mechanical factors are not predominant during biodegradation, they can activate or accelerate it.

### 3.3. Mechanical Properties

The tensile strength and elongation at break of the films of (PVA-b-S)-g-PEGMA as a function of different contents of PVA and S and MWs of PEGMA are presented in Table 4 and Table 5, respectively. It is observed that the tensile strength of the films slightly increased from 17.5 to 23 MPa with an increase in the content of PVA from 5 to 15%, while the tensile strength decreased from 23 to 18 MPa with an increase in the content of starch from 5 to 10%. However, the tensile strength increased from 23 to 28 MPa with an increase in the content of PEGMA from 5 to 10% as shown in Table 4. The increase in the tensile strength of the films is due to hydrogen bonds between PEGMA, S, and PVA [24,39,40,41]. The tensile strength of films of polymers depends on several factors, such as crosslinking, the types of polymers, and the molecular weight of the polymer [30,31]. Table 4 shows that the tensile strength increases with an increase in the molecular weight of PEGMA. M8 (MW 950) showed the highest tensile strength (46, 2 MPa), while M8 (MW 300) showed the lowest tensile strength (28 MPa) with constant contents of PVA, S, and PEGMA. The elongation at break decreased with an increase in the contents of PVA and PEGMA. In addition, elongation at break decreased with an increase in the molecular weight of PEGMA as shown in Table 5. M1 (MW 300) gave the highest elongation at break (150%), while M8 (MW 950) gave the lowest elongation at break (67.5%). From Table 4 and Table 5, the grafting of PEGMA on the blending polymer (PAV-b-S) improved the tensile strength and elongation properties of the films. Similar results based on grafting acrylic acid on the blended polymer (PVA-b-S) are reported [25].

The introduction of crosslinkers caused an increase in film strength, tensile, elongation, and mechanical properties results (inducing a stronger intermolecular force network), which could be attributed to the improvement of the interaction among matrix copolymers (Table 4 and Table 5). Also, mechanical measurements indicated that crosslinking led to varying degrees of film strength and elongation increases. Additionally, the crosslinker mixture performed best in terms of improving the film strength, otherwise the plastic bags would be degraded directly upon immersion in water.

### 3.4. Biodegradability of the Films

Biodegradable polymers (biopolymers, bioplastics) are certified by international regulatory organizations for compliance with international standards: EN 13432 (European) [42], Green PLA (Japanese), ASTM D 6400 (American) [43]. In the certification process, bioplastics undergo multi-stage testing for biodegradation. Only successful results at all stages make it possible to recognize the tested polymer as biodegradable and classify it as a bioplastic. For instance, according to the EN 13432 standard [42], the biodegradation of plastic is tested under standard compost conditions for 180 days. Based on the results of testing bioplastics (in an amount of at least 90%), carbon dioxide, water, and humus should appear. It is considered normal when the original biopolymer leaves behind no more than 10%. At the same time, the resulting compost should not accumulate heavy elements or heavy metals.

The biodegradability of the films of (PVA-b-S)-g-PEGMA with various contents of PVA and S and MWs of PEGMA was studied by immersing the films in a tea solution, soil, tap water, and Coca-Cola for a total time of 120 days. The results in Table 6 show the effect of the tea solution on the biodegradability of the films. As the content of PVA increases in the terpolymer, the degradation day of the films decreases. The hydroxyl groups in the PVA as well as inter- and intra-molecular hydrogen bonding between the hydroxyl groups were ascribed to the decrease in the degradation day of the films [24,39,40]. However, an increase in the MW of the PEGMA in the terpolymer increases the degradation day. The films containing PEGMA with a MW of 300 (M1) degraded for 15 days, while the films containing PEGMA with a MW of 950 (M3) degraded for 20 days. As the S content and MW of PEGMA in the terpolymer increased, the degradation day of the films increased. The time of degradation of the films M4, M5, and M6 is shorter than the time of degradation of the films M1, M2, and M3 as a result of the increased content of S. This is attributed to microbial action towards starch (S), which initiated the initial degradation [41,44]. As shown in Table 6, the content of PEGMA had no effect on the time of degradation of the films.

The time of degradation of the films immersed in soil (Table 7) and in Coca-Cola (Table 8) increases as the contents of PVA and S and MW of PEGMA increase in the terpolymer as shown in Table 7. However, with an increase in the content of S in the terpolymer, there is shorter degradation than PVA and PEGMA in soil but higher degradation in Coca-Cola. This is due to the promotion of biodegradation by microorganisms and enzymes toward starch (S) in the soil [45]. The degradation time of the films in water shows the shortest period compared to the degradation time of the films in the tea solution, soil, and Coca-Cola as shown in Table 9. This is because there is more interaction between the starch and PVA in water than there is in soil, the tea solution, or Coca-Cola [24,45].

### 3.5. Scanning Electron Microscopy (SEM)

**Figure 4:**
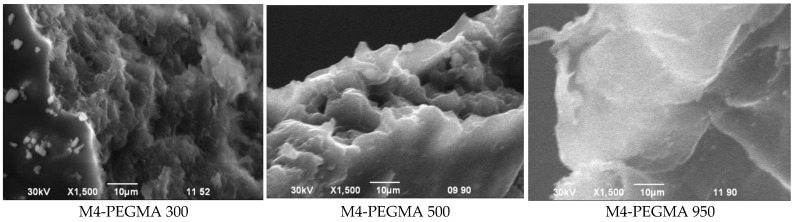
Typical scanning electron microscopy (SEM) image of M4 PEGMA, which is in the size range of 300–950 nm.

Figure 4 shows the morphology of three selected copolymer films, M4-PEGMA 300, M4-PEGMA 500, and M4-PEGMA 950, with varying molecular weights of PEGMA (300, 500, and 950 g/mol), respectively. As the amount of PVA more than doubled, the surface of the film became smoother and shinier. Being a water-soluble synthetic polymer, the increased amount tends to promote a reaction with water vapor in the atmosphere, resulting in a smooth and shiny effect. However, the film sample containing PVA with a higher molecular weight (950 g/mol) does not react with moisture in the atmosphere as easily as the sample containing PVA with a lower molecular weight (300 g/mol). Thus, a rougher surface image of M4-PEGMA 950 than that of M4-PEGMA 500 and M4-PEGMA 300 is depicted in Figure 4.

In more detail, PVA, starch, and PEGMA are not heat sensitive and swell regardless of temperature [46]. When the temperature reaches its lower critical solution temperature (LCST), the components in the polymer composition dehydrate and shrink; thus, a phase separation occurs between the reacting compounds due to different degrees of swelling. Due to shrinkage and phase separation, pores are formed [47]. As for PEGMA 950, due to its high degree of crystallinity, i.e., higher level of crosslinking, swelling is limited, and very few pores are formed. For M4-PEGMA 500 and M4-PEGMA 300, the degree of swelling increased due to the reduced crystallinity, and this contributed to the formation of larger pores. Due to the increase in the molecular weight of PEGMA, the interactions between PVA, S, and PEGMA are enhanced, which limits the mobility of the PEGMA molecules [48]. Thus, very few pores were found in M4-PEGMA 950, resulting in a smooth and shiny effect (Figure 4).

## 4. Conclusions

The effects of PEGMA concentrations and molar weight on the physico-mechanical properties of corn starch/PVA-based films were studied. The addition of PEGMA to the corn starch films increased tensile strength and reduced elongation at break of the blended films. As a result of synthesizing PVA, S, and PEGMA, crosslinked reinforced linkage is formed within the network of the composite. FTIR spectra indicated intermolecular interactions between corn starch/PVA and PEGMA film blends by the H bonds formed (the broadening of the peak in the range from 2500 to 3600 cm^−1^). This type of bond will have an effect on the polymer strength, thermal stability, and mechanical properties, such as tensile strength, where all will be enhanced.

The thermogram analysis of the film blends also confirmed the chemical interaction of corn starch/PVA and PEGMA as ΔH decreases. The homogeneity of the corn starch/PVA and PEGMA films was shown as a single melting peak, and the SEM micrographs visibly confirmed the homogeneous structure of the corn starch/PVA and PEGMA films. All the films stored at room temperature had higher elongation but lower tensile strength than those of the other films. Thus, it seems that the films containing PEGMA 950 had better physical properties than the other films. The time of degradation of the films increase as the contents of PVA and S and MW of PEGMA increase in the terpolymer. We investigated the possibility of using the prepared PVA/S/PEGMA blend films for packing applications. Additionally, we analyzed the alternative tools of getting eco-friendly packaging films and the necessary use of petroleum for preparing polymers. The ability to ‘plant’ polymers of suitable performances from the earth and the environmental problems with plastic pollution were studied.

## Figures and Tables

**Table 1:** The blend copolymer’s PVA and S mix ratios.

	[PVA-b-S] (Proportions)	[PVA-b-S] (mass, g)
M1	1:1	5:5
M2	1.5:1	7:5
M3	2:1	10:5
M4	3:1	15:5
M5	1:2	5:10
M6	3:2	15:10
M7	3:1	15:5
M8	3:1	15:5

**Table 2:** The PEGMA monomer with varied molecular weights (300, 500, and 950 g/mol) and proportions (5, 7, 10, and 15 g).

	[PVA-b-S]	[PVA-b-S]-g-PEGMA300	[PVA-b-S]-g PEGMA500	[PVA-b-S]-g PEGMA950
M1	1:1	1:1:1	1:1:1	1:1:1
M2	1.5:1	1.5:1:1	1.5:1:1	1.5:1:1
M3	2:1	2:1:1	2:1:1	2:1:1
M4	3:1	3:1:1	3:1:1	3:1:1
M5	1:2	3:1.5:1	3:1.5:1	3:1.5:1
M6	3:2	3:2:1	3:2:1	3:2:1
M7	3:1	3:1:1.5	3:1:1.5	3:1:1.5
M8	3:1	3:1:2	3:1:2	3:1:2

**Table 3:** Thermal properties of (PVA-b-S)-g-PEGMA at different MWs of PEGMA.

Copolymer	Temperature Range, °C	Weight Lost %	Residue %	IDT ^a^ °C	PDT_max_^a^ °C	*T_g_*^b^ °C	*T_m_*^b^ °C
M1 300	29.7–386.5	87.4	14.2		210	48.4	190.9
	386.5–531.6	4.40	9.60				
	531.6–896.9	14.6	4.90				
M1 500	30.7–121.8	8.40	90.9	220	310	49.4	195.7
	121.8–182.8	10.6	81.3				
	182.8–261.1	25.8	55.4				
	261.1–381.5	25.3	30.1				
	381.5–524.3	4.80	25.3				
M1 950	29.8–219.1	14.7	85.4	380	500	84.8	209.3
	219.1–275.7	4.50	80.9				
	275.7–333.3	5.60	75.3				
	333.3–438.7	14.2	61.1				
	438.7–899.2	20.3	40.9				

^a^ Determined from TGA curves. ^b^ Determined from DSC curves.

**Table 4:** Tensile strength at break of the films (PVA-b-S)-g-PEGMA increases with an increase in the molecular weight of PEGMA.

Samples	[PVA-b-S]-g-PEGMA	300	500	950
		Tensile, Mpa		
M1	1:1:1	17.5	24.6	34.2
M2	1.5:1:1	18.5	25.7	35.7
M3	2:1:1	20	29.3	37.2
M4	3:1:1	23	33.9	38.9
M5	3:1.5:1	19.5	28.5	37.8
M6	3:2:1	18	25	34.5
M7	3:1:1.5	25.5	30.2	40.1
M8	3:1:2	28	35.7	46.2

**Table 5:** Elongation at break of the films (PVA-b-S)-g-PEGMA decreases with an increase in the molecular weight of PEGMA.

Samples	[PVA-b-S]-g-PEGMA	300	500	950
		Elongation, %		
M1	1:1:1	150	145	134
M2	1.5:1:1	141	138.6	129.3
M3	2:1:1	132	126.77	121.7
M4	3:1:1	128	118.6	110.1
M5	3:1.5:1	145	115.3	110.8
M6	3:2:1	139.5	105.7	101.9
M7	3:1:1.5	120.5	93.8	78.9
M8	3:1:2	105.9	81.6	67.5

**Table 6:** The effect of the tea solution on the biodegradability of the copolymer films.

Samples	[PVA-b-S]-g-PEGMA	300	500	950
		Time of Films Weight Loss until 90% (Days)		
M1	1:1:1	18	18	24
M2	1.5:1:1	17	17	22
M3	2:1:1	15	16	20
M4	3:1:1	12	14	18
M5	3:1.5:1	15	16	18
M6	3:2:1	16	16	20
M7	3:1:1.5	96	100	120
M8	3:1:2	96	100	120

**Table 7:** The effect of soil ground on the biodegradability of the copolymer films.

Samples	[PVA-b-S]-g-PEGMA	300	950	500
		Time of Films Weight Loss until 90% (Days)		
M1	1:1:1	25	30	35
M2	1.5:1:1	25	25	28
M3	2:1:1	30	28	35
M4	3:1:1	18	20	25
M5	3:1.5:1	20	25	28
M6	3:2:1	28	30	36
M7	3:1:1.5	118	120	120
M8	3:1:2	118	120	120

**Table 8:** The effect of Coca-Cola on the biodegradability of the copolymer films.

Samples	[PVA-b-S]-g-PEGMA	300	500	950
		Time of Films Weight Loss until 90% (Days)		
M1	1:1:1	22	26	30
M2	1.5:1:1	25	25	31
M3	2:1:1	28	30	35
M4	3:1:1	30	30	37
M5	3:1.5:1	32	36	43
M6	3:2:1	50	50	56
M7	3:1:1.5	100	120	120
M8	3:1:2	100	120	120

**Table 9:** The effect of water on the biodegradability of the copolymer films.

Samples	[PVA-b-S]-g-PEGMA	300	500	950
		Time of Films Weight Loss until 90% (Days)		
M1	1:1:1	15	16	18
M2	1.5:1:1	16	18	20
M3	2:1:1	16	15	20
M4	3:1:1	8	10	12
M5	3:1.5:1	10	12	15
M6	3:2:1	18	18	22
M7	3:1:1.5	100	120	120
M8	3:1:2	100	120	120

## Data Availability

Raw data will be provided upon request.
